# SIBES: Long-term and large-scale monitoring of intertidal macrozoobenthos and sediment in the Dutch Wadden Sea

**DOI:** 10.1038/s41597-025-04540-9

**Published:** 2025-02-11

**Authors:** Allert I. Bijleveld, Paula de la Barra, Hailley Danielson-Owczynsky, Livia Brunner, Anne Dekinga, Sander Holthuijsen, Job ten Horn, Anne de Jong, Loran Kleine Schaars, Adrienne Kooij, Anita Koolhaas, Hidde Kressin, Felianne van Leersum, Simone Miguel, Luc G. G. de Monte, Dennis Mosk, Amin Niamir, Dorien Oude Luttikhuis, Myron A. Peck, Theunis Piersma, Reyhaneh Roohi, Léon Serre-Fredj, Marten Tacoma, Evaline van Weerlee, Bas de Wit, Roeland A. Bom

**Affiliations:** Department of Coastal Systems, NIOZ Royal Netherlands Institute for Sea Research, NL-1790 AB, Den Burg, Texel, the Netherlands

**Keywords:** Biodiversity, Food webs, Population dynamics, Conservation biology, Zoology

## Abstract

The Wadden Sea is the world’s largest intertidal area and a UNESCO World Heritage Site. Macrozoobenthic invertebrates perform key ecological functions within intertidal areas by regulating nutrient cycles, decomposing organic matter, and providing food for fish, birds and humans. To understand ecological processes and human impacts on biodiversity, the Synoptic Intertidal BEnthic Survey (SIBES) has sampled intertidal macrozoobenthos since 2008. On average 4,109 stations across 1,200 km² of Dutch Wadden Sea mudflats are sampled from June to October to quantify the benthic invertebrate community and sediment composition, including species abundance and biomass, and grain size and mud content. The dataset published now contains 51,851 sampled stations with 3,034,760 individuals of 177 species. This paper details data collection, validation and processing methods. SIBES is ongoing and data will be updated yearly. In sharing these data, we hope to enhance collaborations and understanding of the impact of various pressures on macrozoobenthic invertebrates, sediment composition, food webs, the ecosystem, and biodiversity in the Wadden Sea and other intertidal habitats.

## Background & Summary

Intertidal mudflat ecosystems provide essential foraging, reproduction, and nursery habitat for a variety of organisms including marine mammals, birds, fish, and invertebrates^1,2^. Throughout history, these areas have also provided essential resources for humans^3,4^. Due to the growth of human population size and anthropogenic impact during the last century^4^, many intertidal ecosystems have been lost or are now severely degraded^5^. Intertidal mudflat ecosystems face a multitude of threats, including sea-level rise^6,7^, reduced sediment supply and coastal squeeze^8,9^, warming sea water temperature^10^, wind farm and coastal development^11,12^, aquaculture and fishing practices^13,14^, land subsidence due to salt- and gas mining^15^, compaction of coastal sediments^16^, and eutrophication^17–19^. Many of these threats have an impact on biodiversity and the dynamics of food web and ecosystem processes. Because of the natural value of intertidal ecosystems and their importance to humans, understanding the effects of pressures on these habitats is essential for the effective conservation and sustainable use of these areas.

Central to intertidal mudflat ecosystems are macrozoobenthic invertebrates, i.e. invertebrates larger than 1 mm that live in or near the surface of mudflats. These invertebrates regulate nutrient cycles^20–22^, decompose organic matter^23^, bioturbate^24^, engineer the ecosystem^25–27^, and serve as an important food source for many animal species including fish, birds and humans^28–32^. Additionally, macrozoobenthic invertebrates can be useful indicators of environmental health and human impact. They are relatively sessile and mostly short-lived ( <6 years in most cases)^33^, which limits their ability to escape disturbances and adverse conditions^34^. Due to their sessile nature, the presence of respective species is often correlated with a specific suite of local environmental conditions^35–39^, such as inundation time and sediment composition of the mudflat^35^. If this suite of conditions changes due to human perturbations, it may lead to a shift or disappearance of suitable habitat characteristics, resulting in changes to the macrozoobenthic community. Abundance, species richness, and shifts in community composition of benthic invertebrates and their associated sediments are thus frequently used to assess human disturbances^15,40–45^. Additionally, because benthic invertebrates are important prey for e.g. shorebirds, the implications of human disturbances on their predators can be assessed as well^46–48^.

The Wadden Sea, covering the coastal zones of the Netherlands, Germany and Denmark, is the world’s largest intertidal mudflat ecosystem^49^. The area is recognized as a UNESCO World Heritage Site because of its exceptional natural capital of global importance, including marine mammals, fish and shorebirds^30,49–52^. Despite legal protection and management measures being in place, many economic activities occur in the Dutch Wadden Sea^31,53,54^ that can have detrimental effects on macrozoobenthic invertebrates and their habitat^52^. These activities include commercial fishing for worms^55^, shellfish^42,46,56,57^, and shrimp^58^, as well as mining^15^ that causes (deep) land subsidence^59^. In combination with large-scale phenomena, such as sea level rise^60,61^ and global warming^10,62^, anthropogenic activities can cause habitat alterations and destruction, thus contributing to declines in the abundance of macrozoobenthic invertebrates with effects on the food web and biodiversity^40,52,57,63^. The Wadden Sea has a rich history of research on macrozoobenthic invertebrates, and their habitat use, role in the food web, and the impact of anthropogenic activities^17,64–81^. Sometimes this research is impressively long-term^82^, but it is often focused on a particular area. For a comprehensive understanding, it is also important to study macrozoobenthic invertebrates across a large scale with varying habitats and environmental gradients. Such data are, however, currently lacking.

The Royal Netherlands Institute for Sea Research (NIOZ) has sampled macrozoobenthic invertebrate distributions and sediment composition throughout the Dutch Wadden Sea since 2008. As part of SIBES (Synoptic Intertidal BEnthic Survey), more than four thousand stations distributed across the entire Dutch Wadden Sea have been sampled annually. SIBES is the largest intertidal sampling campaign in the world^83^, and its methodology is now used worldwide, for instance in Portugal, Germany, Australia, Guinea-Bissau, Mauritania, Oman, and China^14,18,84,85^. The spatial sampling performed by SIBES is useful for representative monitoring and for impact assessment studies. The spatial design avoids the problem of selecting control sites for impact assessment, as the entire sampled area can act as a control^86^. With this approach and utilizing the large spatial resolution, it was for example shown that sediment composition and the macrozoobenthic community differed between areas with and without land subsidence^15^. In addition, long-term and large-scale monitoring provides insurance against potentially unmonitored impacts, by providing baseline data for post-hoc comparisons and if unplanned accidents occur^87^.

**Fig. 1:**
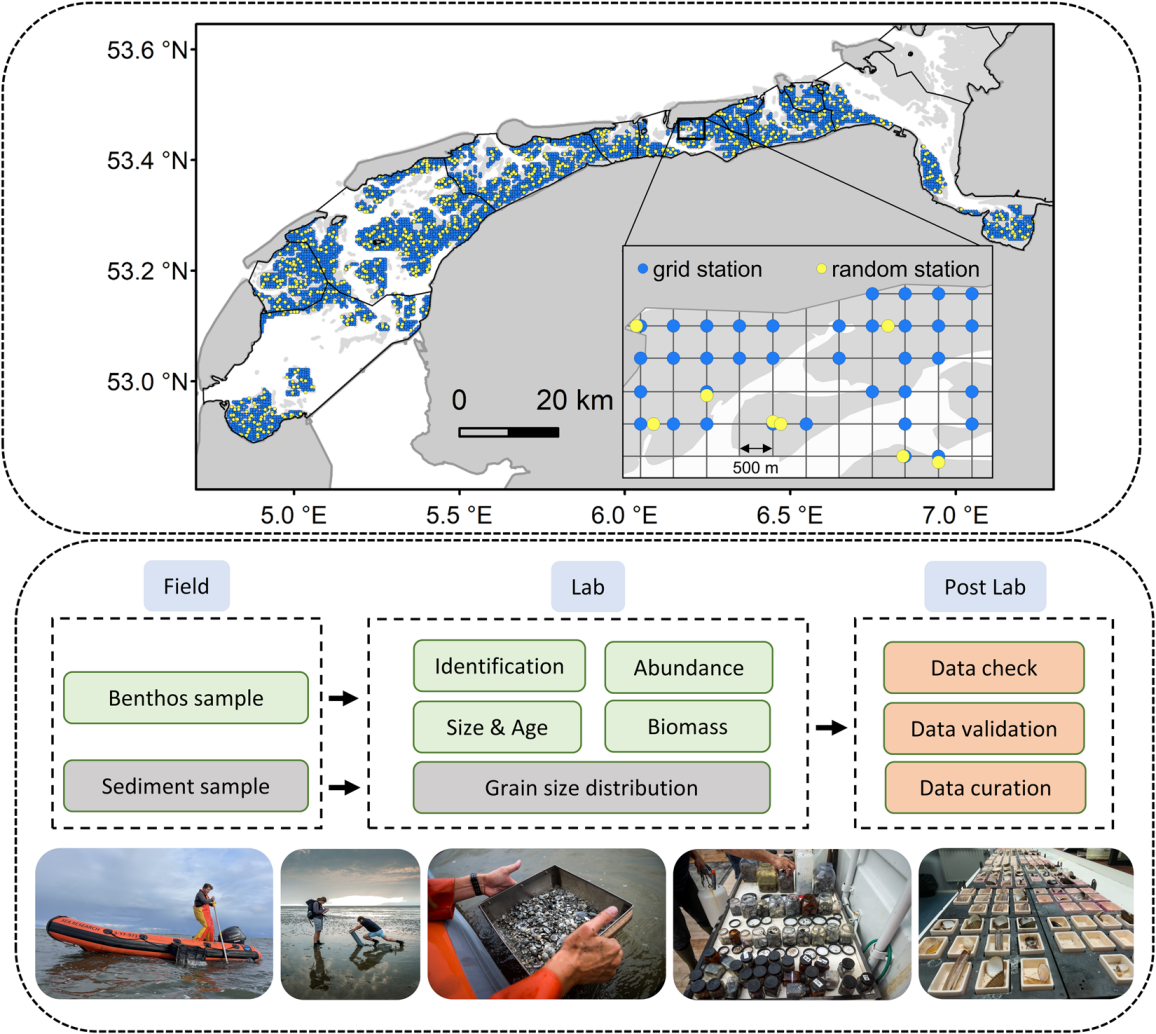
SIBES design with sampling stations and workflow of data collection and processing. The map of the sampling stations shows all stations that have been sampled at least once, with grid (in blue) and random sampling stations (in yellow). The inset shows a map of the detailed design. Tidal basins are separated with black lines. The workflow shows collecting samples in the field, processing them in the laboratory, and curating the data afterwards. Photos from left to right: sampling sediment cores from the boat, sampling by foot, sieved sample, jars with collected samples, and processed samples in crucibles ready for biomass and shell weight measurements in the laboratory. Photos taken by Fred Wiering (left photo) and Kees van de Veen.

To advance fundamental knowledge, buttress collaboration, and assess impacts of human disturbance on intertidal areas, this paper presents SIBES and its continuously growing data set. We describe how the samples are taken in the field and processed in the laboratory, and the procedures for quality assessment and control (Fig. 1). We also describe how the data can be accessed, and how they can be processed within R^88^.

## Methods

### Area description

The Wadden Sea is separated from the North Sea by a series of barrier islands, by which sediment sharing inlet systems connect to back-barrier tidal basins. The area stretches from Den Helder in the Netherlands to Esbjerg in Denmark and spans nearly 500 km linearly following the coast. The total area of the Wadden Sea is about 8000 km^2^ with approximately 50% of this area consisting of intertidal mudflats^89^. These mudflats emerge twice per lunar day around low tide. The Dutch part of the Wadden Sea consists of 10 tidal basins which cover a total area about 2500 km^2^, of which about 50% is intertidal flats^38,52^. This includes the tidal basin Ems-Dollard, the large estuary that borders the German Wadden Sea in the east (Fig. 1).

### Synoptic Intertidal Benthic Survey - SIBES

**Fig. 2:**
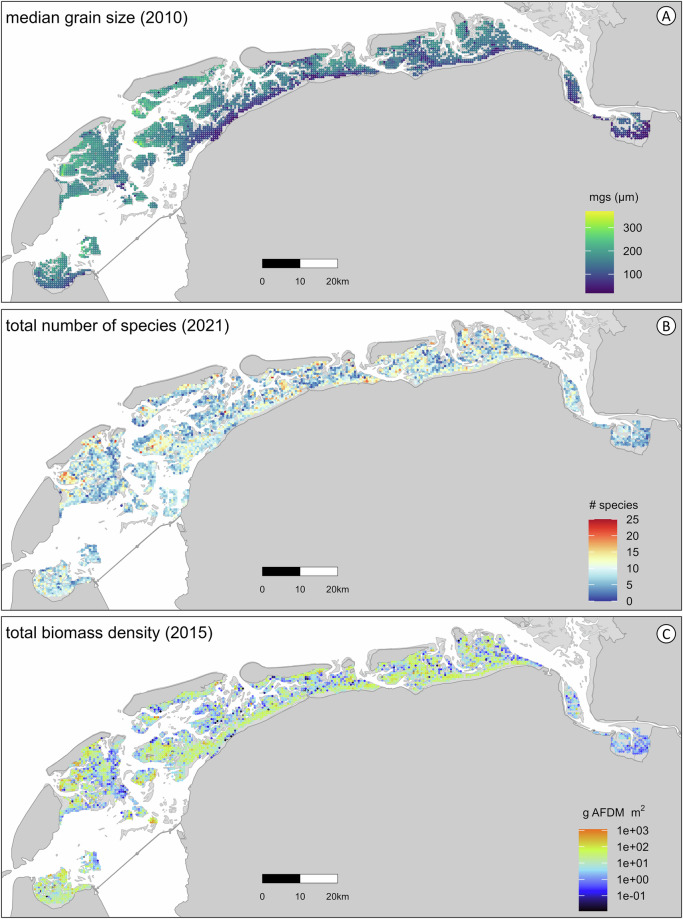
Data examples of the spatial extent and coverage of SIBES. (**A**) Sediment data with median grain size (mgs, in micrometer) per sampling station in 2010. (**B**) Biodiversity as the number of species per sampling station in 2021. (**C**) Total biomass density (g AFDM m^−2^) per sampling station in 2015.

**Fig. 3:**
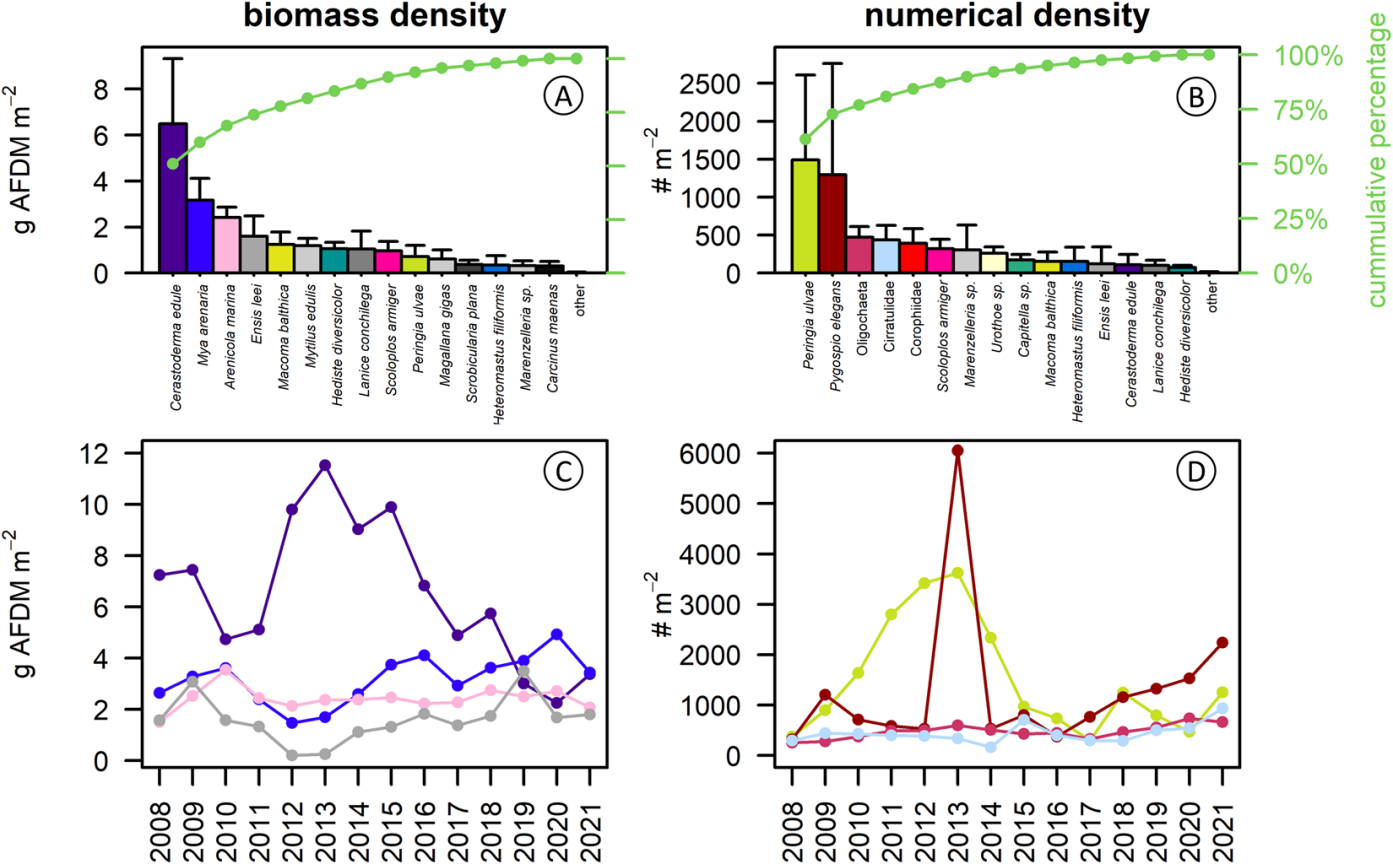
Mean biomass and abundance of benthic invertebrates in the Dutch Wadden Sea. (**A**) Mean annual biomass (g AFDM m^−2^) calculated across 14 years for the 15 most abundant species and all other species summed, ranked from most abundant to least abundant. (**B**) Mean annual numerical abundance (# m^−2^) calculated across 14 years for the 15 most abundant species and all other species summed. (**C**) Total biomass (g AFDM m^−2^) per year for the four most abundant species. (**D**) Total abundance (# m^−2^) per year for the four most abundant species. Error bars in (**A**) and (**B**) denote standard errors of the mean among years. For all species, the colors match between panels.

SIBES is an ongoing ecological monitoring program aimed at monitoring macrozoobenthos and their habitat, i.e. the sediment composition of mudflats (Figs. 2 and 3). Since 2008, every year numerical and biomass density for macrozoobenthic organisms (taxa) are sampled across the entire intertidal Dutch Wadden Sea at a high spatial resolution. These systematic monitoring data have proven fundamental for understanding, for instance, biodiversity^76,90^, distributions and habitat use^69,70,91^, species interactions^23,77,78,92^, the introduction of new species^93^, sediment composition of mudflats^94–97^, and the role of macrozoobenthos in the food web, e.g., as prey to shorebirds^98–100^. In combination with other ecological time-series, for instance on seasonal changes in primary producers^101^ or the distribution of secondary consumers such as shorebirds^102^, these long-term data are invaluable for understanding intertidal ecosystems, and the Wadden Sea in particular. SIBES is also essential for management and evidence-based conservation of the Wadden Sea, such as, in relation to studying the effects of gas extraction^15,103,104^, assessing the European habitat directive^105^, monitoring the quality status of the Wadden Sea^106^, and environmental impact assessments^107^.

### Sampling design

**Fig. 4:**
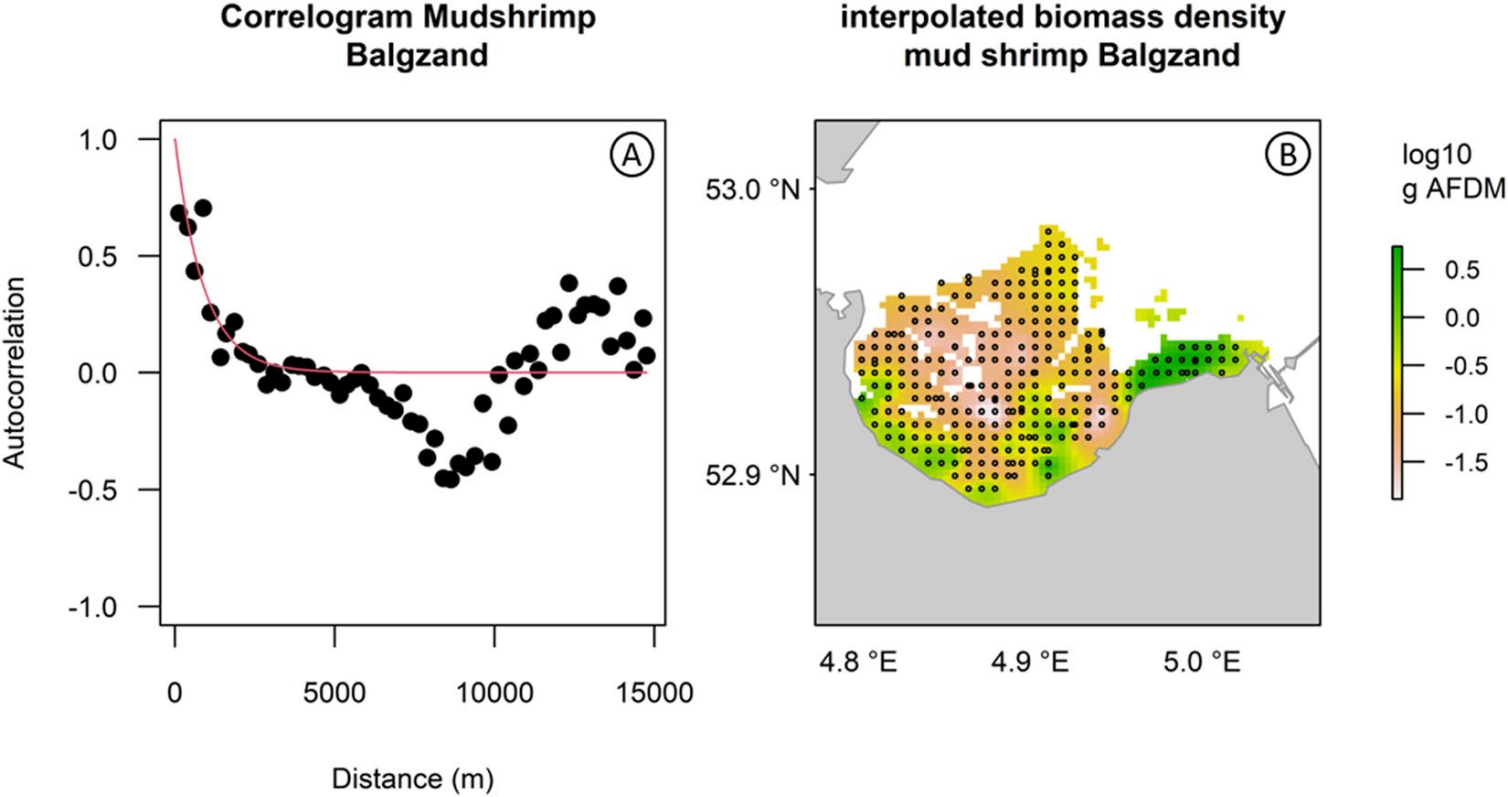
Example of how spatial autocorrelation estimates can be used to interpolate biomass between sampling stations. The spatial sampling design of SIBES with grid and random stations combined allows for accurately estimating autocorrelation. (**A**) The correlogram showing the spatial autocorrelation for Mudshrimp (Corophiidae) at the Balgzand area in 2011. Correlograms can be used to interpolate the abundance of a species between sampling stations as shown in (**B**) where Mudshrimp densities (AFDM m^−2^) were interpolated (mapped) at a 25 × 25 m grid. Actual sampling stations are shown in solid black circles.

The spatial sampling design of SIBES encompasses the entire intertidal Dutch Wadden Sea (Fig. 1) with a combination of a grid sampling (systematic sampling) at an inter-sampling distance of 500 m, and ‘random sampling’ with additional stations placed at random distances from grid sampling stations (hereafter referred to as ‘random stations’)^108^. For logistic convenience, these additional sampling stations were placed on gridlines. To maximize the statistical power to detect changes in abundance of macrozoobenthos, both the grid and random sampling stations were placed at random in the first year and revisited in proceeding years^109^. That is, with a randomly selected starting position, a full 500-m grid was overlaid onto the Wadden Sea. Stations were then removed if they were not located on an intertidal mudflat. The random stations were placed last. The first years of SIBES had approximately 10% random sampling stations, which was increased to almost 20% from 2014 onwards (Table 1) to increase accuracy of estimating spatial autocorrelation parameters^108^. Estimating spatial autocorrelation allows for mapping the distribution of benthic invertebrates^71,98^ (Fig. 4). The Wadden Sea is a dynamic intertidal area in which gullies shift, and mudflats appear and disappear through erosion and sedimentation^110^. Consequently, sampling stations on intertidal mudflats can become too deep to sample. Yearly, sampling stations are removed that are no longer on mudflats and sampling stations are added on the grid if new mudflats arise, while roughly maintaining the percentage of random stations per tidal basin.

**Table 1:** The total number of stations sampled and processed per year.

year	total	Grid	random	% random	% boating	processed
2008	3,892	3,562	330	8	84	3,792
2009	4,067	3,673	394	10	92	3,989
2010	4,051	3,650	401	10	92	3,966
2011	3,947	3,562	385	10	94	3,846
2012	3,920	3,535	385	10	95	3,849
2013	3,981	3,578	403	10	90	3,839
2014	4,216	3,507	709	17	94	4,176
2015	4,219	3,525	694	16	93	4,137
2016	4,278	3,563	715	17	94	4,168
2017	4,231	3,534	697	16	93	2,831
2018	4,233	3,531	702	17	93	2,274
2019	4,270	3,555	715	17	94	4,203
*2020	2,399	1,704	695	29	94	2,366
2021	4,471	3,733	738	17	94	4,415
**averages	4,109	3,565	544	13	92	

The type of sampling station is shown (grid or random) with the percentage of random stations (% random), and the percentage of samples taken by boat instead of by foot (% boating). The numbers of samples processed in the laboratory are also given (processed). Note that sometimes a sample could not be processed in the laboratory and did not lead to an entry in the database (2-3% of the samples taken). Due to lack of funding, not all the samples from 2017 and 2018 have been processed yet.

* Due to COVID-restrictions less samples could be taken.

** Averages are presented without 2020 in which COVID-restrictions were in place.

Overall, between 2,399 and 4,471 stations have been sampled each year (Table 1). In 2008, the Ems-Dollard estuary (the most eastern tidal basin in Fig. 1) was not sampled. In 2020, due to COVID-19 restrictions that limited the crew on board of the research vessel, the grid was sampled at 1000 meters (twice the normal distance), but all random stations were still visited. Excluding 2020, an average of 4,109 stations have been sampled yearly. Between 2017-2018, due to a lack of funding, stations were sampled but not processed in the laboratory. Recent funding has allowed processing these samples that will be finished in 2025. As these data become available, they will be added to the database.

### Field sampling

The NIOZ research vessels RV Navicula (2008–2023) and RV Wim Wolff (from 2024 onwards) were used as a platform to access the sampling areas across the Dutch Wadden Sea. Sampling locations were accessed by rubber dinghies during high-tide or by foot during low-tide (Fig. 1). Because of time constraints and the distances between sampling stations, most samples were taken by boat (Table 1). Sampling stations close to shore were often accessed by foot, as these relatively elevated sites could not be easily reached by boat. Sampling occurred in teams of at least two people and sampling locations were found with a handheld GPS (WGS84 as map datum).

**Fig. 5:**
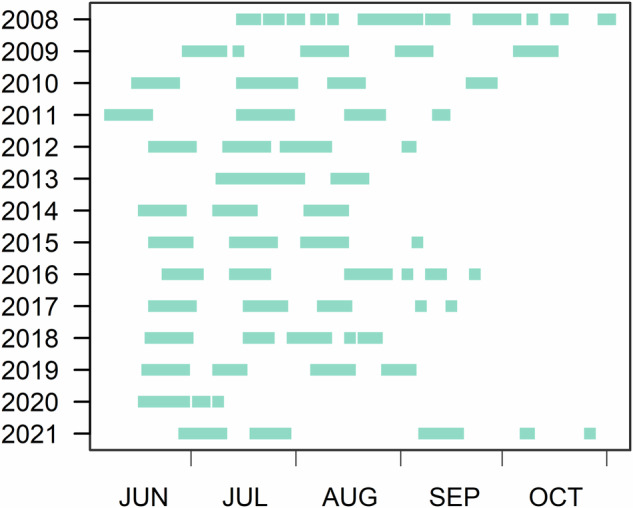
Timing of sampling campaigns for 2008 through 2021. Each green bar represents a sampling period. The sampling period varies somewhat among years due to logistical constraints (including weather) and occurred somewhat later in 2008 and 2021.

In most years, the sampling was conducted for six to eight weeks between June and September (Fig. 5). To prevent an interaction between sampling location and seasonal change (growth of macrozoobenthos), the aim is to sample each area during the same period each year. In 2008, due to logistic constraints, sediment samples were collected on a 1000-m grid instead of at all stations on the 500-m grid.

At each sampling station, a sample was taken by boat or by foot (‘platform’ in Table 2). First, a sediment sample was taken. This was done with a small jar (Polypropylene container) with a diameter of 33 mm to a depth of 4 cm^38,97^. Next, a macrozoobenthic sample was taken. At each site sampled by boat, two cores were taken with a combined area of 0.0173 m^2^ to a depth of ~25 cm. At each site sampled by foot, a single core of 0.0177 m^2^ was taken to a depth of ~25 cm. Both these methods yield similar results (Kraan *et al*., 2007). Details on the sampling cores can be found in the appendix (Supplementary Information S1). In the field, the sample cores were sieved on a 1-mm round mesh (Supplementary Information S1). After sieving, large bivalves (shell length approximately >1 cm) that were easily visible were separated and collected in plastic bags to be frozen at −20 °C for later analysis in the laboratory. The remaining macrozoobenthic species were collected in plastic jars (Fig. 1) and preserved using a 4% formaldehyde solution. To preserve shells and identify live material more easily in the laboratory, all formaldehyde solutions were respectively borax-buffered and stained using rose Bengal dye (C.A.S. no. 632-68-8).

**Table 2:** Description of columns in the file ‘samples.csv’ that contains metadata of all sampling stations as well as data on sediment composition.

SAMPLES.CSV	
column	description
**sample_id**	unique identifier of sample
**sampling_station_id**	unique identifier of sampling station. Sampling stations are repeatedly visited between years
**sampling_type**	grid or random sampling station
**date**	sampling date
**platform**	boat: sample taken from a rubber boat walk: sample taken by foot
**tidal_basin_name**	name of the tidal basin
**tidal_flat_name**	name of the flat as used on Rijkswaterstaat charts
**x**	longitude (decimal degrees east, wgs84)
**y**	latitude (decimal degrees north, wgs84)
**median_grain_size**	median grain size of the sediment in µm
**percentage_mud**	percentage of the sediment sample with grain size < 63 μ*m*

### Laboratory analysis – macrozoobenthic species

Because of the high number of samples processed each year, a trade-off was made between the level of taxonomic detail and the time taken per sample. In practice, this means that all mollusks and larger crustaceans and polychaetes were identified to the species level (Table 3). All other organisms were identified to the finest taxonomical level given in Supplementary Table S1. Identification of the macrozoobenthos species started by using more general keys (e.g. class or order) such as those for Polychaeta by Hartmann-Schröder *et al*.^111^, Amphipoda by Lincoln^112^ and Mollusks by de Bruyne, R. & de Boer, T.W.^113^, and was completed by using recent family or genus keys. The nomenclature was verified in the World Register of Marine Species. Because of time constraints, sponges, hydroids, sea anemones, barnacles, bryozoans, and sea squirts were not analyzed.

**Table 3:** Description of columns in the file ‘species.csv’ that contains information on the species found with SIBES.

SPECIES.CSV	
column	description
**sibes_id**	unique identifier assigned to a specific taxon
**name**	scientific name
**short_name**	abbreviated scientific name
**aphia_id**	identifier of species in WoRMS - World Register of Marine Species: www.marinespecies.org
**taxonomic_group**	taxonomic group: bivalve, crustacea, echinodermata, gastropoda, nemertea, oligochaeta, phoronida, polychaete, polyplacophora, and pycnogonida
**taxonomic_indentification_level**	finest level of guaranteed taxonomic identification
**year_added**	the year the species or group was included
**occurance**	number of samples the species was present
**occupancy**	percentage of samples the species was present
**weight_is_measured**	whether the weight is measured: TRUE or FALSE
**length_measuring_method_id**	method to measure length: 1 = maximum distance on the anterior-posterior axis, 2 = distance between rostrum and tale, 3 = maximum width of carapace, 4 = maximum length of the longest arms, 5 = width of the disc, 6 = max length
**min_shell_length_to_separate_flesh**	minimum shell length to separate shell and flesh for bivalves
**missing_afdm_method_id**	method to predict missing AFDM measurements: 12 = average, 24 = LOESS, 25 = average historical value
**remarks**	remarks on species and taxonomic grouping

After identification, all individuals were counted (Table 4). Polychaetes were often found fragmented and counting them is not straightforward. Between 2008 and into 2011, the number of polychaetes was derived by counting the loose heads and tails. From 2011 onwards, polychaete species were counted by summing the number of heads. For *Lanice conchilega*, only whole individuals were counted.

**Table 4:** Description of columns in the file ‘biota.csv’ that contain abundances and biomass measurements of the species found in the samples.

BIOTA.CSV	
column	description
**sample_id**	unique identifier for the sample, which corresponds with the column ‘sample_id’ in the file SAMPLES.csv
**sibes_id**	taxonomic identifier, which corresponds with the column ‘sibes_id’ in SPECIES.csv
**abundance_m2**	number of individuals per square meter
**afdm_m2**	ash free dry mass (g) per square meter

Note, when a species was absent in a sample, the row is not available. Zero values can be obtained by combining the files ‘biota.csv’ and ‘samples.csv’ by the values in the column ‘sample_id’ (see R-package online). The values ‘abundances_m2’ and ‘afdm_m2’ are standardized for differences in sampled surface area and subsampling. For each species or species group, measurements of individual specimens were grouped per ‘sample_id’ by summation.

For the species and samples that contained many individuals, a subsample was selected and analyzed. The method of subsampling changed in 2014. For the samples collected up to 2013, a subsample was made by dividing the organisms over a sorting tray and a subsample of at least 30 individuals was taken randomly and the subsample percentage noted down. For the samples collected in 2014 and onwards, a subsample was taken by dividing the organisms equally over the middle 4 bands of the sorting tray. A subsample of around 30 individuals was taken. A dice was thrown to decide where in the tray the subsample was taken. Subsamples were taken for *Peringia ulvae*, small bivalves, Crustaceans and small worms (including Oligochaeta) which can all occur at high densities.

The body size of species was measured according to the methods in Supplementary Table S1. All crustaceans were identified, but because of time constraints lengths were only taken for shrimps and crabs. All individuals were measured to the nearest mm using a vernier or a digital caliper. For *Macoma balthica* and *Cerastoderma edule* shells larger than 8 mm, the width and thickness were also measured, where possible. Occasionally, length could not be measured, for instance, because a shell was broken. In those cases and when possible, length was estimated. The bivalve *Ensis leei* would often break and the length could not be measured. In such cases the length (mm) was estimated with the width (mm) of the top or bottom following: length = 6.5 × width_top_ or 6.6 × width_bottom_.

The biomass of individuals was measured as Ash Free Dry Mass (AFDM) (Table 4), that was determined by first drying the sample for 2 to 3 days at 60 °C in a ventilated stove, then measuring dry mass. Following this, the sample was incinerated for 5 hours at 560 °C and then the mass was measured again to calculate AFDM. Measurements of mass were done by hand until July 2011. From July 2011 onwards, most measurements of mass were made by the WULC automatic weighing machine. Only very large crucibles were still weighed by hand. Shell and meat were separated before AFDM was measured, but only for individuals above a minimum size (see Supplementary Table S1 for the thresholds). Bivalves below the minimum size threshold were incinerated together. For worms, biomass was determined for all species in 2008 and 2009. To allow processing many samples, from 2010 onwards, biomass was not measured for the small worms *Pygospio elegans, Spio martinensis, Cirratulidae* (*Aphelochaeta sp*. and *Tharyx sp*.), and Oligochaeta.

### Laboratory analysis – sediment samples

The sediment samples were first freeze-dried for up to 96 h, then homogenized with a mortar and pestle. The mass of homogenized samples was measured, and the sample was mixed with degassed water, purified by means of reverse osmosis, and placed into 13-ml polypropylene auto-sampler tubes. Samples were then shaken vigorously with a vortex mixer for 30 s prior to grain sizes being measured using a particle size analyzer that uses laser diffraction and polarization intensity differential scattering technology (Coulter LS 13 320, optical module ‘grey’). Grain sizes were measured from 0.04 to 2000 μ*m* in 126 size classes. From these distributions, median grain size and mud content (volume percentage of particles <63 μ*m*) were extracted (Table 2). Organic matter and calcium carbonate were not removed by treating sediment samples with hydrogen chloride and hydrogen peroxide^38^.

### Data validation and processing

**Fig. 6:**
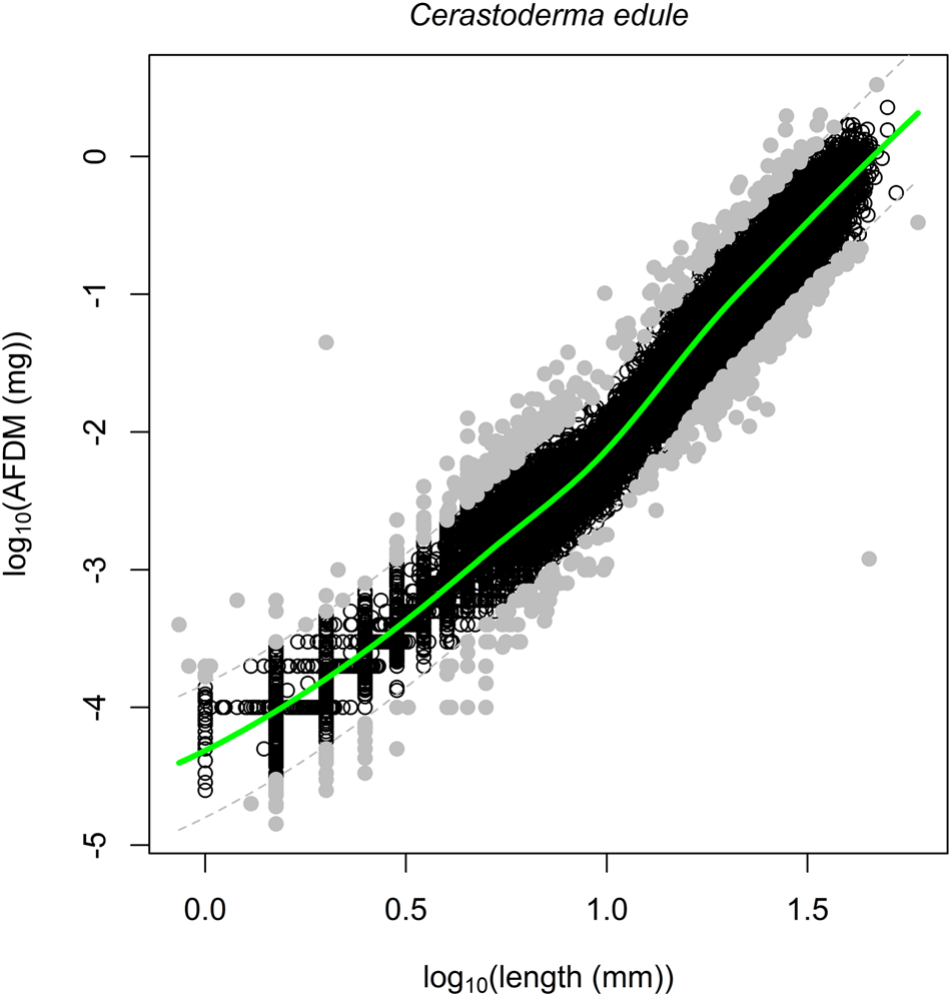
Example of a species-specific allometric relationship used for predicting an individual’s Ash Free Dry Mass (AFDM, g) from (estimated) length (mm). The solid green line is the fitted LOESS-model that is used for predicting AFDM from length. The LOESS curve was fitted using the ‘loess’ function in R^88^, with a span of 0.6. The model’s residual standard error was 0.15. Open circles are individual measurements (n = 58,989) and grey solid dots are outliers (n = 560; corresponding to 0.9% of the data points) that fall outside of twice the inter quartile range.

After processing samples in the laboratory, data records were checked for quality through, e.g., outlier analyses. To identify erroneous biomass measurements, for example, biomass was analyzed against length to ensure they followed plausible and understandable patterns of variability (Fig. 6). Extreme abundances, biomass and length values were double checked for mistakes and corrected or deleted if the mistake was unclear. For animals without recorded lengths, boxplots were created to identify outliers, which were then reviewed for potential data entry errors and corrected if necessary. The lengths of bivalves were checked against maximal length values reported in de Bruyne & de Boer (2008)^113^.

In some cases, AFDM measurements could not be made, for instance because the shell was broken, or values were deleted after they were identified as outliers. If a length measure was available, AFDM was predicted from allometric relationships (Fig. 6). Because allometric relationships can be non-linear, they were modelled using LOESS^71^. Because these biomass-length relationships can differ between years and areas, we used a hierarchical approach for each species or species group (Supplementary Table S1). That is, we first filtered the data for that species or group per season, then per year, then per region, and then per tidal basin. After each step, the number of records with biomass and length measures was determined. The LOESS-model was then fitted at the smallest scale that included at least 75 individual records of biomass and length measured. For species and species groups <11 mm, 45 individual records were sufficient. When no length value was available to predict AFDM for a species or species group, the average biomass was used following the same hierarchical approach to get the most fine-grained input possible, but with a minimum of 30 individual records.

All total abundance (#) and biomass (g AFDM) measurements were standardized per unit area (m^−2^) by dividing them by the sampled surface area. In the case of subsampling (see section ‘*Laboratory analysis – macrozoobenthic species’*), abundances were corrected by dividing the densities with the fraction of the subsample. After this standardization, individual measurements were grouped per species or species group and summed per sample (‘sample_id’, Table 2). The underlying individual measurement data as well as raw sediment distribution data are available, but because they are sensitive for erroneous calculations, they are not shared here but available upon request.

## Data Records

Here, we present the currently available SIBES data that spans 14 years (2008–2021). SIBES has continued beyond these years and those data will also be made available in the future (see the section “Usage Notes”). With 5,107 unique sampling stations (‘sampling_station_id’ in Table 2), the dataset currently includes 51,851 sampled stations (‘sample_id’ in Table 2) with biomass and abundance of 177 taxa (‘sibes_id’ in Table 3) and 48,186 of sediment composition. The measurements of biomass and abundance presented here were based on 3,034,760 individuals. These data are available in three ‘csv’ files that can be downloaded from the NIOZ Data Archive System DAS^114^. The files contain (1) metadata of sampling stations and sediment characteristics (‘sample.csv’, Table 2), (2) species found on the mudflats of the Dutch Wadden Sea (‘species.csv’, Table 3), and (3) biomass and abundance per species per sample station (‘biota.csv’, Table 4).

## Technical Validation

Before starting SIBES in 2008, a simulation study^108^ was conducted to estimate the optimal sampling design for different (sometimes conflicting) objectives while maximizing statistical power and efficiency of sampling. Building on existing intertidal benthic monitoring programs in the Dutch Wadden Sea^64,83,115,116^, three objectives were identified: (1) estimating population sizes, monitoring of population trends, and comparisons of populations/trends between years or areas, (2) mapping species distributions, and (3) accurately estimating spatial autocorrelation parameters, which is required for mapping abundances. Four commonly used sampling designs were compared: simple random, grid, and two types of transects. An additional fifth novel design was also included: grid with random replacements. Sampling designs were analyzed using Monte Carlo simulations at four levels of naturally occurring spatial autocorrelation. To compare sampling designs, the following criteria were used: (1) minimum detectable difference in mean between two time periods or two areas, (2) mean prediction error and (3) estimation bias of autocorrelation parameters. It was shown that grid sampling with a number of random samples was accurate and effective, and the optimal sampling design catering for all three objectives. Therefore, when SIBES started in 2008, the optimal design of combined grid sampling with a percentage of random stations was chosen. In the simulation study, these random samples were replaced to maintain equal samples sizes and a fair comparison between sampling designs. With SIBES, due to logistic efficiency in the field, these were additional stations placed on gridlines (Fig. 1).

To further validate the SIBES sampling scheme, data were compared with traditional transect sampling that occurred in Balgzand, the western most tidal basin of the Wadden Sea^64,82^. These analyses indicated that standardized (m^−2^) abundance and biomass estimates were, on average, similar but with two striking differences^117^: i) SIBES showed larger accuracy with lower variance around the estimates, and ii) the densities of deeper living species, such as *Mya arenaria* and *Arenicola marina*, were underestimated with SIBES. A potential cause of the latter difference could be that sampling depth is reduced when a rubber boat is needed to access and sample the station.

To continuously ensure a high and standardized data quality, sampling and processing are conducted with protocols in accordance with NEN-EN-ISO 9001:2015. Since 2010, the SIBES quality management system externally evaluated (Kiwa) each year according to ISO 9001 standards.

## Usage Notes

Because SIBES is ongoing, the dataset will be updated, and data will be made available online yearly^114^. To allow ongoing research to be completed (e.g. PhD-candidates publishing their work), there is a delay of three years after data collection. However, the most recent data are also available upon request. Likewise, the underlying data of individual measurements as well as raw sediment distribution are available upon request. When using these data, people are urged to cite this data paper in any resulting product (e.g. publication or report). These products and collaborations will help us to maintain this valuable monitoring program that requires substantial effort and resources. More information and documentation on SIBES can be found online (www.nioz.nl/sibes).

## Supplementary information

Supplementary Table S1

Supplementary Information S1

## Data Availability

To aid the use and exploration of SIBES data using the software package R^88^, we have developed an R-library that will continue to be updated. The latest version can be accessed on: https://github.com/allertbijleveld/SIBES. With data included in the R-library, examples are provided for preparing the data, exploring trends, and making maps.
