# Supplementary material for: SIBES: Long-term and large-scale monitoring of intertidal macrozoobenthos and sediment in the Dutch Wadden Sea
Source: Sci Data. 2025 Feb 11;12:239. doi: 10.1038/s41597-025-04540-9 (PMC11814293; doi:10.1038/s41597-025-04540-9)
Supplement: Supplementary file 2 — Supplementary Information S1 [file 41597_2025_4540_MOESM2_ESM.pdf]

SAMPLING CORE FOR WALKING

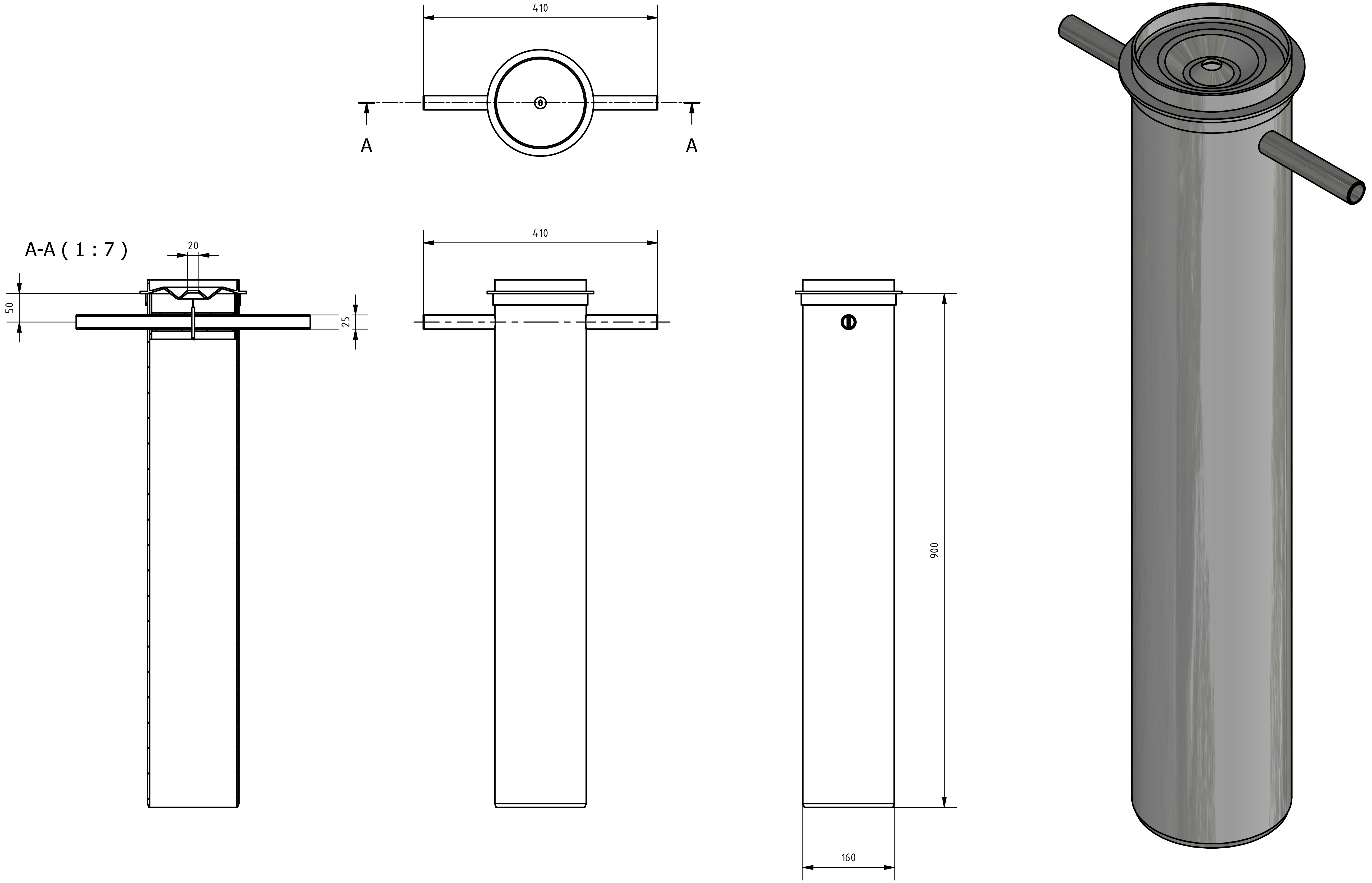

Amerikaanse projectie

Schaal:
Maateenheid: mm
Datum:

Getekend: Edwin
Afdeling: Development
Gezien:

Opmerkingen:
mat. PVC

National Marine Facilities

Benaming: SIBES walking core

Nummer: 1
Van: 1

Formaat: A3

SAMPLING CORE FOR BOATING
Original design by Anne Dekinga

REVISIE TABEL			
Stuknr.	Omschrijving	Materiaal	Gecontroleerd

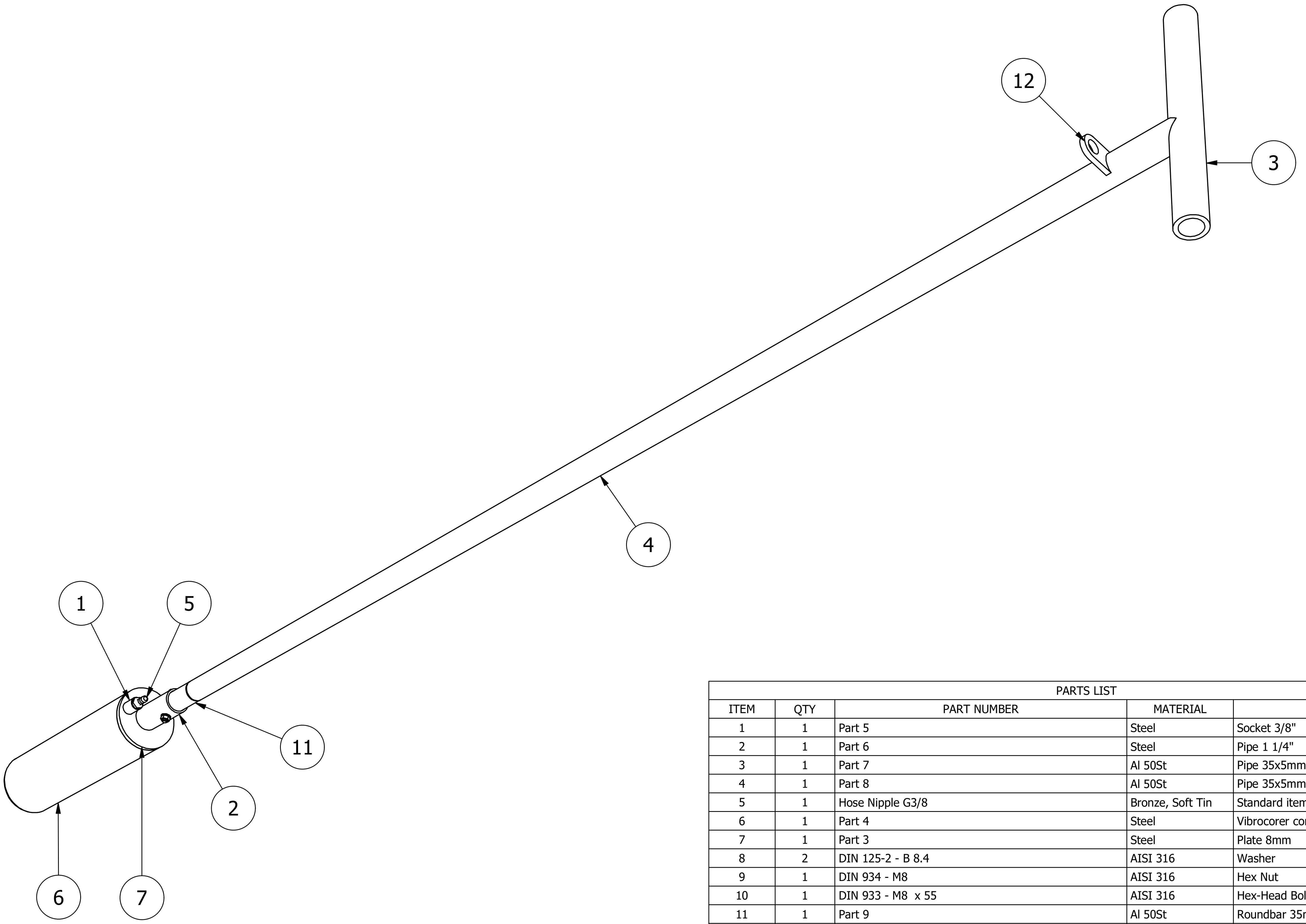

PARTS LIST

ITEM	QTY	PART NUMBER	MATERIAL	DESCRIPTION
1	1	Part 5	Steel	Socket 3/8"
2	1	Part 6	Steel	Pipe 1 1/4"
3	1	Part 7	Al 50St	Pipe 35x5mm
4	1	Part 8	Al 50St	Pipe 35x5mm
5	1	Hose Nipple G3/8	Bronze, Soft Tin	Standard item
6	1	Part 4	Steel	Vibrocorer coretube
7	1	Part 3	Steel	Plate 8mm
8	2	DIN 125-2 - B 8.4	AISI 316	Washer
9	1	DIN 934 - M8	AISI 316	Hex Nut
10	1	DIN 933 - M8 x 55	AISI 316	Hex-Head Bolt
11	1	Part 9	Al 50St	Roundbar 35mm
12	1	Holder	Al 50St	Flatbar 6mm

Amerikaanse projectie	Schaal: 1:6	Getekend: L.Boom	Opmerkingen:
	Maateenheid: mm	Afdeling: NMF-Support	
	Datum: 07-09-2017	Gezien:	
NIOZ Technology Marine	Benaming: SIBES boating core	Nummer: 1 Van: 3	Formaat: A3

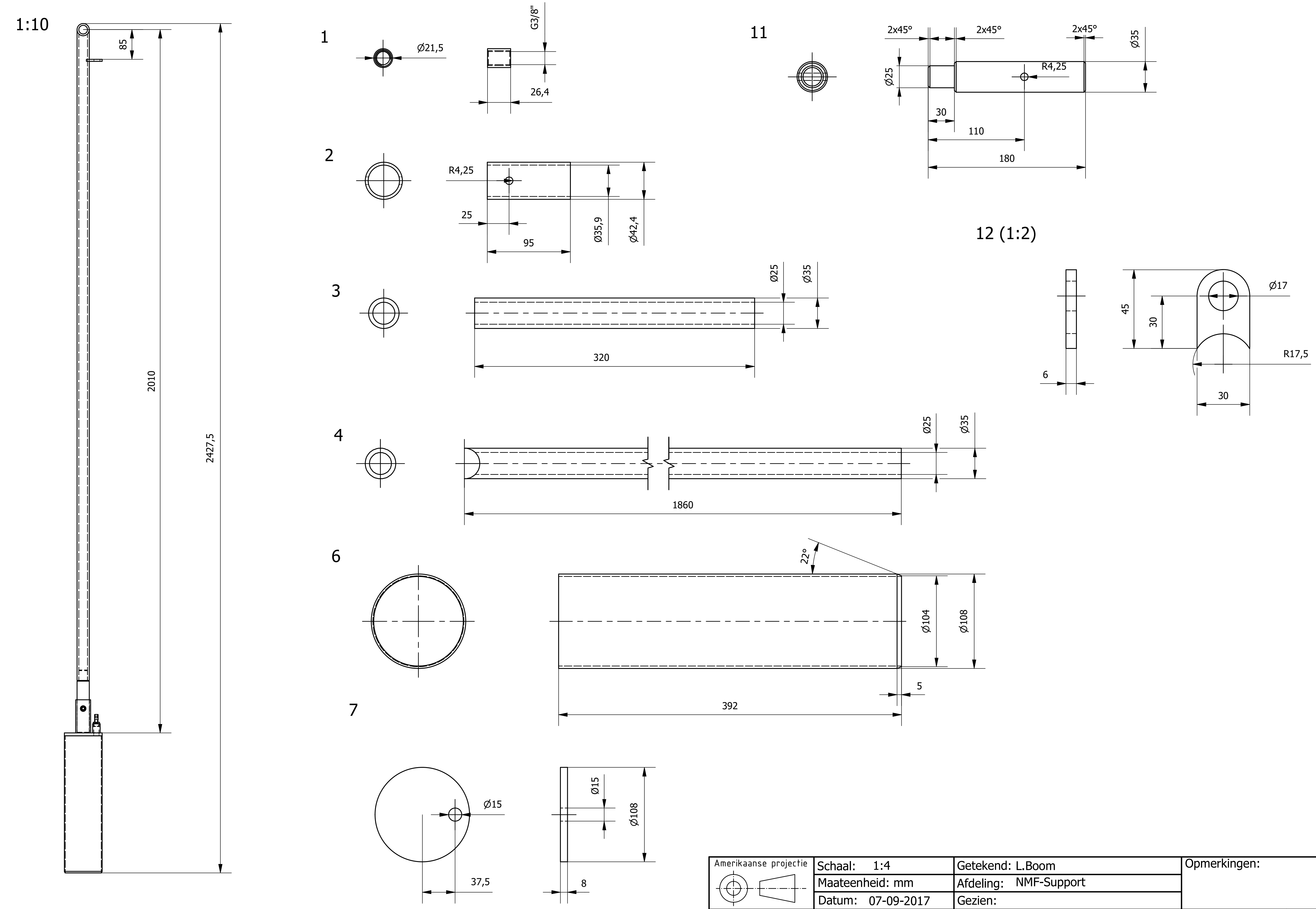

Amerikaanse projectie	Schaal: 1:4	Getekend: L.Boom	Opmerkingen:
	Maateenheid: mm	Afdeling: NMF-Support	
	Datum: 07-09-2017	Gezien:	
NIOZ Technology Marine	Benaming: SIBES boating core	Nummer: 2 Van: 3	Formaat: A3

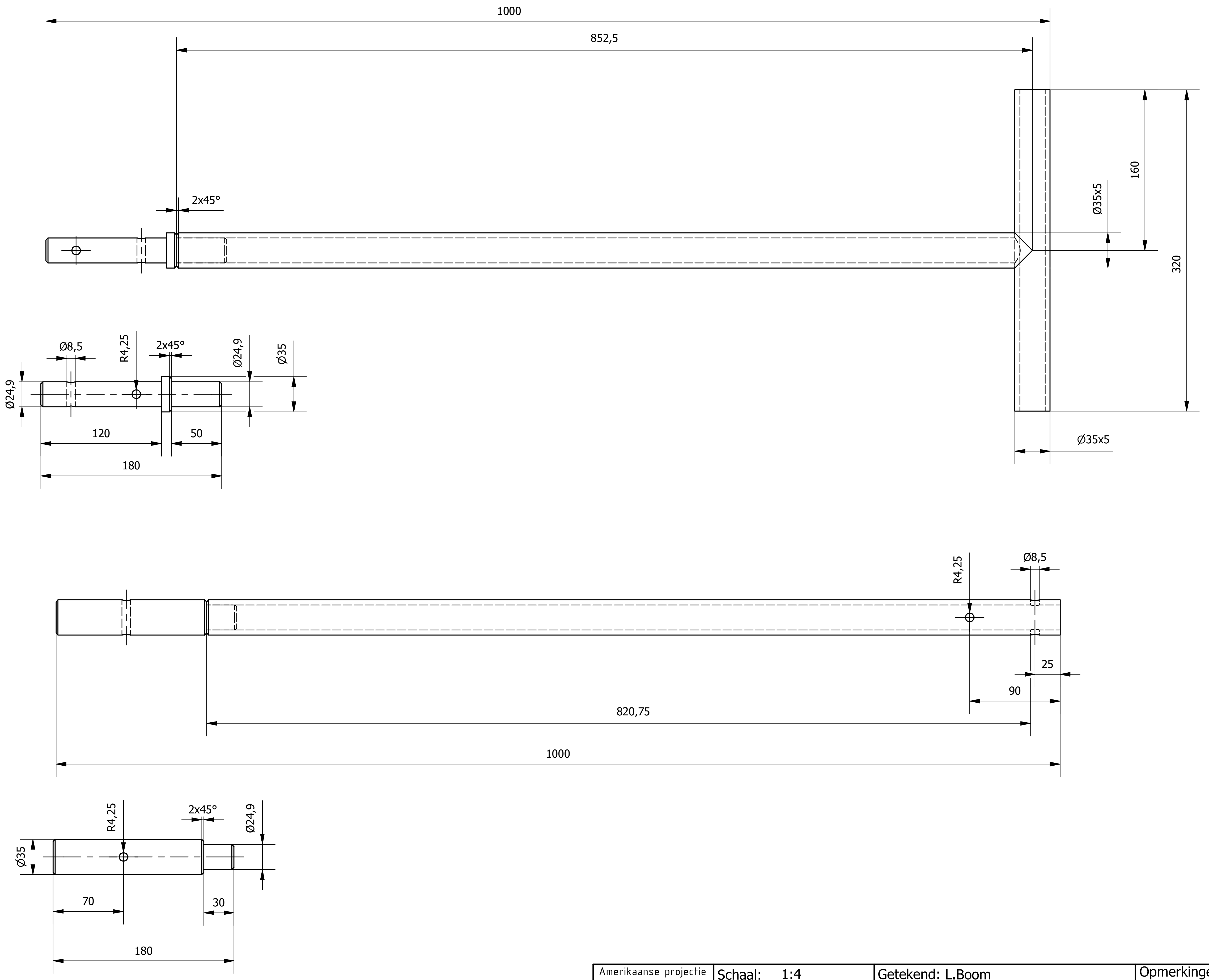

Amerikaanse projectie	Schaal: 1:4	Getekend: L.Boom	Opmerkingen:
	Maateenheid: mm	Afdeling: NMF-Support	
	Datum: 10-03-2020	Gezien:	
NIOZ Marine Technology	Benaming: SIBES boating core	Nummer: 3 Van: 3	Formaat: A3

SAMPLING SIEVE
Original design by Anne Dekinga

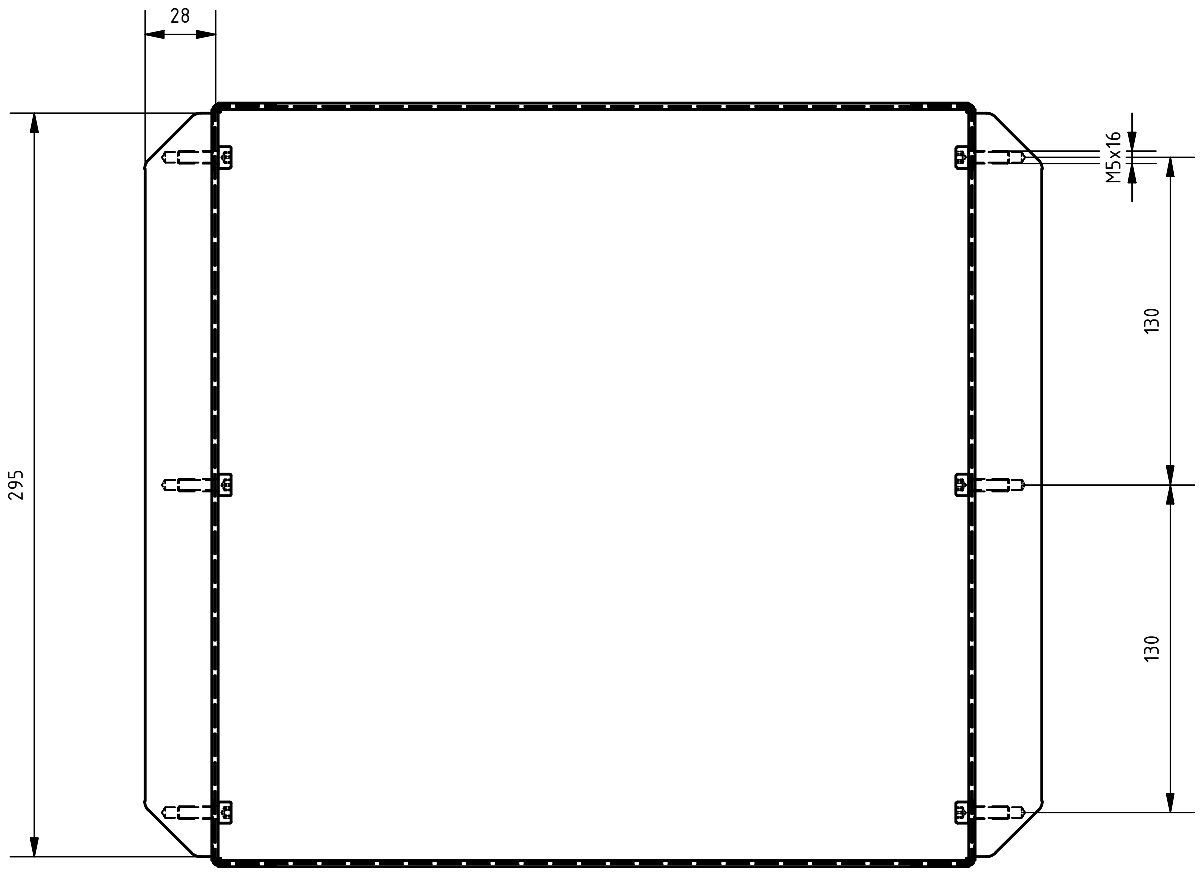

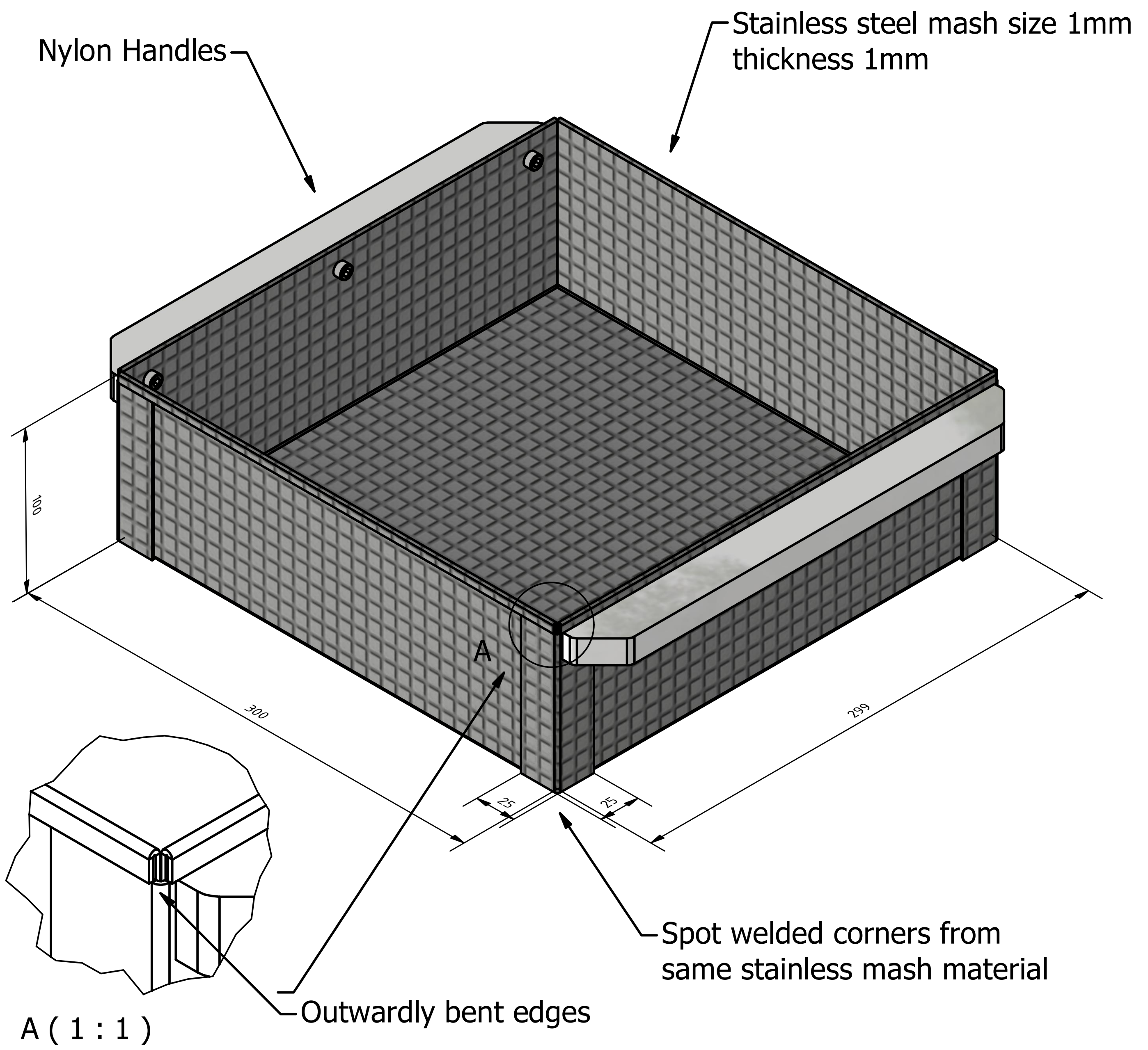

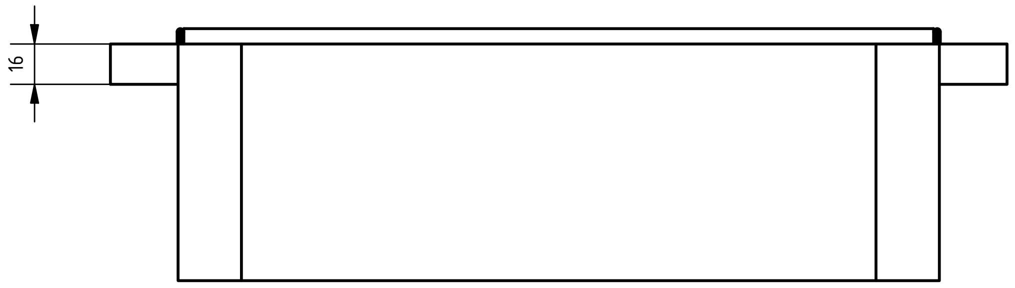

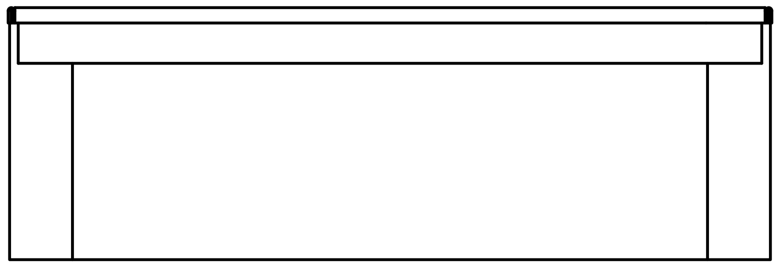

Opslaglocatie: L:\development\mti\Inventor tekeningen\Sediment zeef\Sediment zeef.idw			
Amerikaanse projectie	Schaal:	Getekend: Edwin	Opmerkingen: material: Stainless Steel mash 1mm
	Maateenheid: mm	Afdeling: Development	
	Datum:	Gezien:	
National Marine Facilities	Benaming: Sediment Sieve	Nummer: 1 Van: 1	Formaat: A3
